# Observer-Based Time-Variant Spacing Policy for a Platoon of Non-Holonomic Mobile Robots

**DOI:** 10.3390/s21113824

**Published:** 2021-05-31

**Authors:** Martín Velasco-Villa, Raúl Dalí Cruz-Morales, Alejandro Rodriguez-Angeles, Carlos A. Domínguez-Ortega

**Affiliations:** 1 CINVESTAV-IPN Electrical Engineering Department, Mechatronics Section, Av. I.P.N. No. 2508, Col. San Pedro Zacatenco, Mexico City 07360, Mexico; velasco@cinvestav.mx (M.V.-V.); carlos.dominguez@cinvestav.mx (C.A.D.-O.); 2 UNAM FES Cuautitlán, Engineering Department, Electrical Engineering Section, Carretera Cuautitlán-Teoloyucan Km. 2.5, San Sebastián Xhala, Cuautitlán Izcalli 54714, Mexico; rdcruz@comunidad.unam.mx

**Keywords:** platoon formation, input-delay observer, non-holonomic mobile robots, time-varying spacing policy

## Abstract

This paper presents a navigation strategy for a platoon of *n* non-holonomic mobile robots with a time-varying spacing policy between each pair of successive robots at the platoon, such that a safe trailing distance is maintained at any speed, avoiding the robots getting too close to each other. It is intended that all the vehicles in the formation follow the trajectory described by the leader robot, which is generated by bounded input velocities. To establish a chain formation among the vehicles, it is required that, for each pair of successive vehicles, the (i+1)-th one follows the trajectory executed by the former *i*-th one, with a delay of τ(t) units of time. An observer is proposed to estimate the trajectory, velocities, and positions of the *i*-th vehicle, delayed τ(t) units of time, consequently generating the desired path for the (i+1)-th vehicle, avoiding numerical approximations of the velocities, rendering robustness against noise and corrupted or missing data as well as to external disturbances. Besides the time-varying gap, a constant-time gap is used to get a secure trailing distance between each two successive robots. The presented platoon formation strategy is analyzed and proven by using Lyapunov theory, concluding asymptotic convergence for the posture tracking between the (i+1)-th robot and the virtual reference provided by the observer that corresponds to the *i*-th robot. The strategy is evaluated by numerical simulations and real-time experiments.

## 1. Introduction

There are several mobile robot applications that take advantage of platooning strategies to improve performance or because of safety issues. Either at street vehicles or small mobile robot applications, a platoon is formed by a leading vehicle and a known group of follower vehicles that may not be aware of all the members that make up the squad, or all the information that comes from them, but usually each robot has information only from its predecessor.

The control of vehicle platoons has led to several approaches to deal with this problem, highlighting the follower vehicle scheme and the cooperative adaptive cruise control (CACC) [1,2]. Although, the control actions for a dynamic model of a vehicle (acceleration and steering) are different from a kinematic perspective (linear and rotational velocities), the strategies to render platoon formation are basically the same, and can be extrapolated to any type of mobile robot. The main goal is to ensure that the vehicles form a chain, maintaining a separation between them, dictated by a spacing policy, either distance or time based [3,4,5,6,7,8,9,10]. In addition, string stability has to be guaranteed by assuring the attenuation of the effects of disturbances, caused by initial conditions, speed variations, or external disturbances, along the vehicle string [5,11,12,13].

As mentioned, platoon formation can be seen as a leader–follower problem considering pairs of successive vehicles at the chain, such as the framework presented in [14], where the leader–follower formation is converted into a trajectory tracking control problem, with the aim to keep a desired constant distance and a bearing angle between the follower and leader robots; the proposed control strategy considers both, the kinematic and dynamic models of a differential mobile robot, particularly of the *TurtleBot*. Simulation and experimental results show tracking of a desired trajectory for a triangle formation, however, it is observed that keeping a distance and bearing angle between leader and follower robots prevents the follower from performing exactly the same path of the leader, which is more obvious when moving into a curve, since the follower would develop a parallel curve with respect to the leader, or even an over steer or under steer curve depending on whether the follower is in the inner or outer side of the curve. Similar results are shown in [15], where a dispersed structure is forced as a formation to a group of non-holonomic mobile robots, such virtual structure is kept by defining relative distances and angles between the robots in the group. The aforementioned works are a type of constant spacing policy since they enforced constant distance and bearing angles between the robots in the formation. Other works based on the constant spacing policy are for instance [3,4,16,17,18,19]. As mentioned before, among vehicle platooning, there is a large number of works devoted to solving the longitudinal control of vehicles, without considering the lateral control [18,20,21], furthermore, either a punctual mass system or double integrator dynamics is commonly assumed, such as in [17,19,20], where platoon control is proposed to ensure a prescribed performance.

Several works under a time spacing scheme have been carried out in the past few years, see for instance [3,4,5,12,20,22]. A constant distance and time spacing policy are considered in [3] for platoons of differential drive mobile robots by using odometry information. In [12], a constant headway time is designed to obtain a graceful degradation of one-vehicle look-ahead CACC based on estimating the preceding vehicle’s acceleration. A delay-based spacing policy has been designed in [5] for the control of vehicle platoons considering disturbances and the string stability of the approximated model of the vehicles. Another delay-based spacing policy can be seen in [23,24], where an approach based on an input-delay observer to obtain a fixed time-gap separation is used for the differential drive of a mobile robot platoon.

It has been pointed out that a time spacing policy improves traffic efficiency compared to constant distance separations [12], because if the platoon moves at a slow speed, it will not be necessary to maintain a large distance between vehicles, which makes the string inefficient. For this reason, the use of variable separations as a function of the speed of the platoon is a method to improve performance. In a time spacing policy, most approaches consider that the velocity of the platoon should not approach zero, since if it is reduced, the vehicle separation will also approach zero and there could be a collision between the vehicles. For this reason, the present paper proposes an extension of the work developed in [23,24] where a fixed time-delay spacing policy is proposed, broadening the work to a time-varying spacing policy inspired by artificial potential fields, which ensures a safe distance between the members of the string, avoiding robots from getting too close to each other. Under these conditions, the work considers a squad of non-holonomic mobile robots, where each robot is intended to follow the position and orientation of its precedent vehicle in the formation, while maintaining a time-varying gap that avoids collisions when the robots approach each other. To accomplish this task, the (i+1)-th robot will follow the path of the *i*-th robot, delayed by a time-varying gap, so an input-delay observer that estimates the trajectory to be executed by each (i+1)-th robot is proposed, rendering robustness to the platoon against external disturbances. The presented scheme is proven by Lyapunov stability theory. The performance of the strategy was evaluated by numerical simulations and real-time experiments.

The rest of the document continues in Section 2, presenting the problem formulation associated with a set of *n* mobile robots type (2,0), represented by kinematic models. Further, the proposal of the time-varying spacing policy is analyzed. In Section 3, the design of an input-delayed observer to generate desired trajectories is presented, and in Section 4, the proposed navigation strategy is described in detail. Section 5 is devoted to the evaluation of the presented time-varying spacing policy through numerical evaluation and real-time experiments. A discussion of the performance of the proposed strategy based on the numerical simulation and experimental results is presented in Section 6. Finally, Section 7 presents the conclusions of the work.

## 2. Problem Formulation

**Figure 1:**
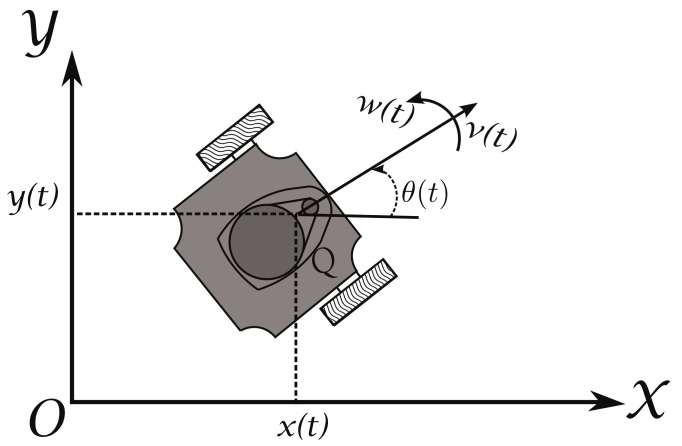
Differential driven mobile robot.

Take into account a set of *n* differential driven mobile robots that have two actuated wheels and move on the X−Y plane, as the one shown in Figure 1. The position, at time *t*, of the point *Q*, located at the midpoint of the robot’s wheels axis with respect to the global coordinate frame $O_{xy}$ is denoted by the coordinates x(t) and y(t), while the orientation is denoted by θ(t). The kinematic model of this robot can be obtained by the geometric interrelations shown in Figure 1 [25]; obtaining for the *i*-th robot,

(1) $$\begin{array}{ccc} {\dot{x}}_{i}\left(t\right) & = & \nu_{i}\left(t\right)\cos\left(\theta_{i}\left(t\right)\right)\\ {\dot{y}}_{i}\left(t\right) & = & \nu_{i}\left(t\right)\sin\left(\theta_{i}\left(t\right)\right)\\ {\dot{\theta }}_{i}\left(t\right) & = & \omega_{i}\left(t\right) \end{array}$$

where ${\left[x_{i}\left(t\right),y_{i}\left(t\right),\theta_{i}\left(t\right)\right]}^{T}$ is the state vector of the system and $u_{i}\left(t\right)={\left[\nu_{i}\left(t\right),\omega_{i}\left(t\right)\right]}^{T}$ are the input signals, in which, $\nu_{i}\left(t\right)$ is the linear or translational velocity and $\omega_{i}\left(t\right)$ is the angular velocity, with i=1,2,3,⋯,n. It is assumed that the robot is a rigid mechanism that ideally moves on a flat surface, without friction, and must move only by the velocities exerted by the wheels, the vertical axes of the wheels are perpendicular to the ground where it moves, and the non-holonomic constraint,

(2) $${\dot{x}}_{i}\left(t\right)\sin\left(\theta_{i}\left(t\right)\right)-{\dot{y}}_{i}\left(t\right)\cos\left(\theta_{i}\left(t\right)\right)=0$$

is satisfied at all times.

### 2.1. Platoon Formation

**Figure 2:**
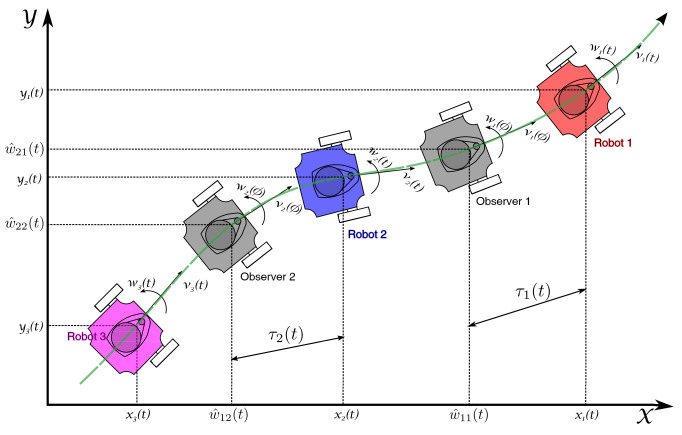
Time-varying gap formation strategy.

For the set of *n* wheeled mobile robots, a platoon formation problem is taken into consideration in which the first robot (i=1, leader of the formation), performs any trajectory produced by bounded input velocities $\nu_{i}\left(t\right)$, $\omega_{i}\left(t\right)$, this is, only the leader knows the path to be performed by the platoon. Note that in the case of a vehicle car platoon, this assumption is satisfied by considering that the leader vehicle is driven by a human operator, who sets the traveling route. The platoon consists of a string formation topology, where the (i+1)-th robot, for i=1,⋯,n−1, will perform the trajectory of the *i*-th robot that is delayed by a specific time-varying gap $\tau_{i}\left(t\right)$, to maintain a time-varying spacing policy between successive robots. To obtain the delayed trajectory of the *i*-th robot in the formation, an input-delay observer is designed based on the position measurements of the *i*-th robot. The considered time-varying strategy is shown in Figure 2, where a convoy of three robots and two observers is presented. It is intended that the (i+1)-th robot tracks the path provided by the *i*-th observer, for i=1,⋯,n−1, maintaining the formation, while avoiding getting too close to each other.

### 2.2. Spacing Policy

When considering a time spacing policy in a string formation, the platoon performance is improved with respect to fixed distance spacing policies [12], since in a time-gap strategy, the distance between any pair of successive robots is varying depending on the velocity of the members of the chain, decreasing for slow velocities and increasing otherwise. This strategy is intuitively applied by most human drivers when the speed of the vehicles decreases, for instance when vehicles approach a pedestrian crossing line or a red traffic light, where it is not necessary to keep a large inter-vehicle distance. Notice that in a fixed time-gap strategy reducing the traveling velocity may produce a collision situation, since the inter-vehicle distance also decreases. To avoid such a collision scenario, in this work, a time-varying spacing policy, inversely proportional to the distance between vehicles, is proposed based on the fixed-time spacing policy presented in [24]. It is considered a time-varying gap $\tau_{i}\left(t\right)$ that increases its magnitude when the distance between vehicles reaches a threshold, which implies that the velocity of the formation is too slow. For this time-varying gap, the following assumption is taken into account.

The time-varying gap is proposed as,

(3) $$\tau_{i}\left(t\right)=\tau_{s_{i}}+\tau_{c_{i}}\left(t\right)$$

where $\tau_{s_{i}}>0$ is a fixed time-gap, which keeps a distance among each pair of successive vehicles at the platoon, which also varies according to the velocity; $\tau_{c_{i}}\left(t\right)$ is a time-varying gap that increases when the *i*-th and the (i+1)-th robot approximate each other, rendering a safe distance. That is, $\tau_{c_{i}}\left(t\right)$ adds a varying distance to the one induced by $\tau_{s_{i}}>0$, and this varying distance is activated when slow velocities render a separation distance smaller than a desired magnitude.

**Figure 3:**
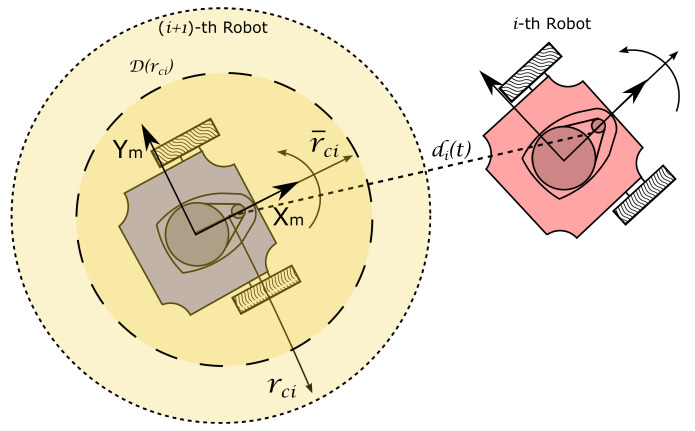
Time-varying gap configuration between two successive robots.

The time-varying gap $\tau_{i}\left(t\right)$ should be a bounded non-negative differentiable function, whose value tends to increase as the (i+1)-th robot approaches to the *i*-th robot. Inspired by [26], the time-varying component $\tau_{c_{i}}\left(t\right)$ is proposed as,

(4) $$\tau_{c_{i}}\left(t\right)=\left\{\begin{array}{cc} \frac{\alpha_{i}}{2}{\left(\frac{1}{r_{c_{i}}}-\frac{1}{d_{i}\left(t\right)}\right)}^{2} & \mathrm{if}\;{\bar{r}}_{c_{i}}\leq d_{i}\left(t\right)\leq r_{c_{i}}\\ 0 & \mathrm{otherwise} \end{array}\right.$$

where ${\bar{r}}_{c_{i}}$ and $r_{c_{i}}$ are positive, non-zero constants that determine the zone where $\tau_{c_{i}}\left(t\right)$ is defined, around the (i+1)-th robot, see Figure 3. The parameter $\alpha_{i}$ is a constant of proportionality and $d_{i}\left(t\right)$ is the Euclidean distance between the position of the *i*-th and (i+1)-th robot, this is,

(5) $$d_{i}\left(t\right)=\sqrt{{\left(x_{i}\left(t\right)-x_{i+1}\left(t\right)\right)}^{2}+{\left(y_{i}\left(t\right)-y_{i+1}\left(t\right)\right)}^{2}}.$$

Using the Euclidean distance is conservative with respect to the distance between two successive vehicles along the path, nevertheless is rather easier to be determined by using onboard sensors, as well as by transmitted positions between the vehicles. Meanwhile, safe trailing distance and assured clear distance ahead (ACDA) are implicitly satisfied when considering standard distance between vehicles.

Note that the definition of $\tau_{ci}\left(t\right)$, implies that it is bounded by below by zero. Thus, when the distance between vehicles is bigger than the desired safe distance, $d_{i}\left(t\right)>r_{c_{i}}$, the separation distance is the only function of the constant-time gap $\tau_{s_{i}}$. Otherwise, when the separation distance between vehicles becomes smaller than the safe distance, the time-varying component $\tau_{c_{i}}\left(t\right)$ tends to increase its magnitude to produce the desired safe distance. As a matter of fact, the behavior of the time-varying component $\tau_{c_{i}}\left(t\right)$ increases and decreases the distance separation gap generated by the positive limits of $d_{i}\left(t\right)$, this is, ${\bar{r}}_{c_{i}}\leq d_{i}\left(t\right)\leq r_{c_{i}}$.

The time derivative of the time-varying gap is given by ${\dot{\tau }}_{i}\left(t\right)={\dot{\tau }}_{c_{i}}\left(t\right)$, this is,

(6) $${\dot{\tau }}_{i}\left(t\right)=\left\{\begin{array}{cc} \left(\frac{\alpha_{i}}{r_{c_{i}}}-\frac{\alpha_{i}}{d_{i}\left(t\right)}\right)\left(\frac{{\dot{d}}_{i}\left(t\right)}{d_{i}^{2}\left(t\right)}\right) & \mathrm{if}\;{\bar{r}}_{c_{i}}\leq d_{i}\left(t\right)\leq r_{c_{i}}\\ 0 & \mathrm{otherwise} \end{array}\right.$$

with,

(7) $${\dot{d}}_{i}\left(t\right)=\frac{a_{1i}+a_{2i}}{\sqrt{{\left(x_{i}\left(t\right)-x_{i+1}\left(t\right)\right)}^{2}+{\left(y_{i}\left(t\right)-y_{i+1}\left(t\right)\right)}^{2}}}$$

where $a_{1i}=\left(x_{i}\left(t\right)-x_{i+1}\left(t\right)\right)\left({\dot{x}}_{i}\left(t\right)-{\dot{x}}_{i+1}\left(t\right)\right)$ and $a_{2i}=\left(y_{i}\left(t\right)-y_{i+1}\left(t\right)\right)\left({\dot{y}}_{i}\left(t\right)-{\dot{y}}_{i+1}\left(t\right)\right)$.

Notice that the time-varying gap $\tau_{i}\left(t\right)$ will increase its magnitude affecting the behavior of the (i+1)-th robot, only when the *i*-th robot gets inside the influence zone $D(r_{c_{i}})$ defined over the mobile robot frame $X_{m_{i}}-Y_{m_{i}}$ as,

$$D\left(r_{c_{i}}\right)=\left\{q_{m_{i}}={\left[x_{m_{i}}\;\;y_{m_{i}}\right]}^{T}\in R^{2}\left|{\bar{r}}_{c_{i}}\leq ∥q_{m_{i}}∥\leq r_{c_{i}}\right.\right\}$$

of the (i+1)-th robot, avoiding collisions between them, as depicted in Figure 3. The values of $r_{c_{i}}$ and ${\bar{r}}_{c_{i}}$ of the constant time-gap $\tau_{s_{i}}$ must be chosen according to the size of the robot and the desired working conditions, particularly according to the desired safe distance, and the maximum rejection action, settled by the inner radio ${\bar{r}}_{c_{i}}$, which for zero value would imply an infinite action.

**Remark** **1.** *It is important to point out that $\tau_{i}\left(t\right)$, in (3), is a bounded function independently of the position of the i-th and (i+1)-th robots, this is, $\tau_{i}\left(t\right)$ will be bounded since,*

$$0\leq \tau_{ci}\left(t\right)\leq \frac{\alpha_{i}}{2}{\left(\frac{{\bar{r}}_{c_{i}}-r_{c_{i}}}{r_{c_{i}}{\bar{r}}_{c_{i}}}\right)}^{2}$$

*where the upper bound of $\tau_{ci}\left(t\right)$ could increase to infinity in the case that ${\bar{r}}_{c_{i}}$ tends to zero. Notice also that by the definition of $\tau_{ci}\left(t\right)$, from (6), ${\dot{\tau }}_{i}\left(t\right)$ will be also a bounded function. Therefore, the time-varying gap $\tau_{i}\left(t\right)$ and its time derivative ${\dot{\tau }}_{i}\left(t\right)$ are bounded ∀t>0, in its region of definition, this is, $sup\left\{\tau_{i}\left(t\right)\right\}\leq {\bar{\tau }}_{i}$ and $sup\left\{{\dot{\tau }}_{i}\left(t\right)\right\}\leq \mu_{i}$ with ${\bar{\tau }}_{i},\mu_{i}\in R_{+}$.*

**Remark** **2.** *To determine the parameters involved in the influence zone $D(r_{c_{i}})$, the following simple heuristic steps are proposed:*

*(i)*  *Determine ${\bar{r}}_{c_{i}}$ depending on the physical structure of the robots.*
*(ii)*  *Propose the size of the influence zone by setting $r_{c_{i}}$ such that ${\bar{r}}_{c_{i}}\leq r_{c_{i}}$.*
*(iii)*  *Determine $\alpha_{i}$ such that the increment produced on $\tau_{i}\left(t\right)$ avoids a possible collision between involved successive robots. This parameter depends on the possible velocity of the robots.*

## 3. Input-Delayed Observer

With the aim of estimating the delayed trajectory accomplished by the *i*-th robot, an input-delay observer is designed to provide the delayed position, orientation, and velocities of the *i*-th robot based on current measurements. The following assumptions are taken into account.

**Assumption** **1.** *The posture $\left[x_{i}\left(t\right),y_{i}\left(t\right),\theta_{i}\left(t\right)\right]$ of the i-th robot is available for measurement ∀t>0.*

**Assumption** **2.** *The input signals of the leader robot of the platoon (i=1), $\left[\nu_{1}\left(t\right),\omega_{1}\left(t\right)\right]$ are bounded ∀t>0, this is $sup\left\{\nu_{1}\left(t\right)\right\}\leq \bar{\nu_{1}}$ and $sup\left\{\omega_{1}\left(t\right)\right\}\leq \bar{\omega_{1}}$ with $\bar{\nu_{1}},\bar{\omega_{1}}\in R_{+}$. Thus, a feasible trajectory to be followed by the robots at the platoon is generated.*

Associated to the *i*-th robot, it is possible to define a time-varying function,

(8) $$\phi_{i}\left(t\right)=t-\tau_{i}\left(t\right)$$

and the set of $\tau_{i}\left(t\right)$ units of time delayed variables,

(9) $$\begin{array}{cc} w_{1_{i}}\left(t\right) & =x_{i}\left(\phi_{i}\right)=x_{i}\left(t-\tau_{i}\left(t\right)\right)\\ w_{2_{i}}\left(t\right) & =y_{i}\left(\phi_{i}\right)=y_{i}\left(t-\tau_{i}\left(t\right)\right)\\ w_{3_{i}}\left(t\right) & =\theta_{i}\left(\phi_{i}\right)=\theta_{i}\left(t-\tau_{i}\left(t\right)\right) \end{array}$$

where it is assumed that $t>\tau_{i}\left(t\right)$.

Considering the kinematic model of the *i*-th robot (1), the time derivatives of the delayed states (9) are,

$$\begin{array}{cc} {\dot{w}}_{1_{i}}\left(t\right) & ={\dot{x}}_{i}\left(\phi_{i}\right)=\frac{\partial x_{i}}{\partial \phi_{i}}\frac{\partial \phi_{i}}{\partial t}=\nu_{i}\left(\phi_{i}\right)\cos\left(\theta_{i}\left(\phi_{i}\right)\right){\dot{\phi }}_{i}\left(t\right)\\ {\dot{w}}_{2_{i}}\left(t\right) & ={\dot{y}}_{i}\left(\phi_{i}\right)=\frac{\partial y_{i}}{\partial \phi_{i}}\frac{\partial \phi_{i}}{\partial t}=\nu_{i}\left(\phi_{i}\right)\sin\left(\theta_{i}\left(\phi_{i}\right)\right){\dot{\phi }}_{i}\left(t\right)\\ {\dot{w}}_{3_{i}}\left(t\right) & ={\dot{\theta }}_{i}\left(\phi_{i}\right)=\frac{\partial \theta_{i}}{\partial \phi_{i}}\frac{\partial \phi_{i}}{\partial t}=\omega_{i}\left(\phi_{i}\right){\dot{\phi }}_{i}\left(t\right) \end{array}$$

where,

(10) $${\dot{\phi }}_{i}\left(t\right)=1-{\dot{\tau }}_{i}\left(t\right).$$

The dynamics of the *i*-th mobile robot (1) considering a $\tau_{i}\left(t\right)$ units of time delay, can be rewritten as,

(11) $$\begin{array}{cc} {\dot{w}}_{1_{i}}\left(t\right) & ={\dot{\phi }}_{i}\left(t\right)\nu_{i}\left(\phi_{i}\right)\cos\left(w_{3_{i}}\right)\\ {\dot{w}}_{2_{i}}\left(t\right) & ={\dot{\phi }}_{i}\left(t\right)\nu_{i}\left(\phi_{i}\right)\sin\left(w_{3_{i}}\right)\\ {\dot{w}}_{3_{i}}\left(t\right) & ={\dot{\phi }}_{i}\left(t\right)\omega_{i}\left(\phi_{i}\right). \end{array}$$

Since the time-varying delayed dynamics (11) is a free delayed system, it is possible now to propose a nonlinear Luenberger-type observer with the intention that the estimated state converges to the one of the delayed dynamics (11). The proposed observer takes the form,

(12) $$\begin{array}{cc} {\dot{\hat{w}}}_{1_{i}}\left(t\right) & ={\dot{\phi }}_{i}\left(t\right)\nu_{i}\left(\phi_{i}\right)\cos\left({\hat{w}}_{3_{i}}\right)+\lambda_{1_{i}}e_{w_{1i}}\left(t\right)\\ {\dot{\hat{w}}}_{2_{i}}\left(t\right) & ={\dot{\phi }}_{i}\left(t\right)\nu_{i}\left(\phi_{i}\right)\sin\left({\hat{w}}_{3_{i}}\right)+\lambda_{1_{i}}e_{w_{2i}}\left(t\right)\\ {\dot{\hat{w}}}_{3_{i}}\left(t\right) & ={\dot{\phi }}_{i}\left(t\right)\omega_{i}\left(\phi_{i}\right)+\lambda_{2_{i}}e_{w_{3i}}\left(t\right) \end{array}$$

where $\lambda_{1_{i}},\lambda_{2_{i}}\in R_{+}$ and $e_{w_{1i}}\left(t\right),e_{w_{2i}}\left(t\right)$ y $e_{w_{3i}}\left(t\right)$ are the observation errors defined as,

(13) $$\begin{array}{cc} e_{w_{1i}}\left(t\right) & =w_{1_{i}}\left(t\right)-{\hat{w}}_{1_{i}}\left(t\right)\\ e_{w_{2i}}\left(t\right) & =w_{2_{i}}\left(t\right)-{\hat{w}}_{2_{i}}\left(t\right)\\ e_{w_{3i}}\left(t\right) & =w_{3_{i}}\left(t\right)-{\hat{w}}_{3_{i}}\left(t\right). \end{array}$$

Note that the observation errors $e_{w_{ji}}\left(t\right)$ depend on the delayed variable $w_{j_{i}}\left(t\right)=x_{i}\left(t-\tau_{i}\left(t\right)\right)$ that has to be injected to the observer. Due to the fact that the *i*-th robot state (1) can be measured (Assumption 1), the injection error in (12) is obtained by storing a segment of the trajectory of the *i*-th robot (1).

### Convergence of the Observation Errors

In order to facilitate the analysis of the convergence properties of the observation errors (13), instead of considering the original inertial representation of the observer (12), the body frame representation is obtained by means of a rotation of the observation error in the form,

(14) $$\left[\begin{array}{c} \epsilon_{1_{i}}\left(t\right)\\ \epsilon_{2_{i}}\left(t\right)\\ \epsilon_{3_{i}}\left(t\right) \end{array}\right]=\left[\begin{array}{ccc} \cos w_{3_{i}}\left(t\right) & \sin w_{3_{i}}\left(t\right) & 0\\ -\sin w_{3_{i}}\left(t\right) & \cos w_{3_{i}}\left(t\right) & 0\\ 0 & 0 & 1 \end{array}\right]\left[\begin{array}{c} e_{w_{1i}}\left(t\right)\\ e_{w_{2i}}\left(t\right)\\ e_{w_{3i}}\left(t\right) \end{array}\right]$$

this is,

(15) $$\begin{array}{cc} \epsilon_{1_{i}}\left(t\right)= & e_{w_{1i}}\left(t\right)\cos w_{3_{i}}\left(t\right)+e_{w_{2i}}\left(t\right)\sin w_{3_{i}}\left(t\right)\\ \epsilon_{2_{i}}\left(t\right)= & -e_{w_{1i}}\left(t\right)\sin w_{3_{i}}\left(t\right)+e_{w_{2i}}\left(t\right)\cos w_{3_{i}}\left(t\right)\\ \epsilon_{3_{i}}\left(t\right)= & e_{w_{3i}}\left(t\right). \end{array}$$

Taking the time derivative of the observation errors (15), it is obtained,

(16) $$\begin{array}{cc} {\dot{\epsilon }}_{1_{i}}\left(t\right)= & -\lambda_{1_{i}}\epsilon_{1_{i}}\left(t\right)\\ \; & +{\dot{\phi }}_{i}\left(t\right)\left[2\nu_{i}\left(\phi_{i}\right)\sin^{2}\left(\frac{\epsilon_{3_{i}}\left(t\right)}{2}\right)+\omega_{i}\left(\phi_{i}\right)\epsilon_{2_{i}}\left(t\right)\right]\\ {\dot{\epsilon }}_{2_{i}}\left(t\right)= & -\lambda_{1_{i}}\epsilon_{2_{i}}\left(t\right)\\ \; & +{\dot{\phi }}_{i}\left(t\right)\left[\nu_{i}\left(\phi_{i}\right)\sin\left(\epsilon_{3_{i}}\left(t\right)\right)-\omega_{i}\left(\phi_{i}\right)\epsilon_{1_{i}}\left(t\right)\right]\\ {\dot{\epsilon }}_{3_{i}}\left(t\right)= & -\lambda_{2_{i}}\epsilon_{3_{i}}\left(t\right). \end{array}$$

The convergence properties of the observation errors are formally presented in the following lemma.

**Lemma** **1.** *Consider that the i-th robot satisfies Assumptions 1 and 2. Then, if $\lambda_{1_{i}},\lambda_{2_{i}}>0$, the states ${\hat{w}}_{j_{i}}$ and their time derivatives ${\dot{\hat{w}}}_{j_{i}}$, for j=1,2,3, given by the observer (12), present exponentially convergence to the trajectory of the i-th robot delayed $\tau_{i}\left(t\right)$ units of time.*

**Proof.**  Suppose that leader of the platoon, robot (i=1), satisfies Assumption 1 and 2, and assume that the second robot (i=2) follows the delayed trajectory of the leader robot, provided by the state of the time-varying delayed observer (12).

If robot 2 follows the delayed trajectory of robot 1, it is evident that robot 2 must have a set of bounded inputs that allows it to follow the desired trajectory. Assuming that the preceding arguments are valid until the (i−1)-th robot, it is possible to consider that for the *i*-th robot, taking into consideration that for $\lambda_{2_{i}}>0$,

(17) $${\dot{\epsilon }}_{3_{i}}\left(t\right)=-\lambda_{2_{i}}\epsilon_{3_{i}}\left(t\right)$$

exponentially converges to zero, then, the proof is reduced to demonstrate the convergence of errors $\epsilon_{1_{i}}\left(t\right)$ and $\epsilon_{2_{i}}\left(t\right)$.

Defining,

(18) $${\bar{\epsilon }}_{i}\left(t\right)={\left[\begin{array}{cc} \epsilon_{1_{i}}\left(t\right) & \epsilon_{2_{i}}\left(t\right) \end{array}\right]}^{T}$$

it is possible to write,

(19) $${\dot{\bar{\epsilon }}}_{i}\left(t\right)=A_{i}\left(t\right){\bar{\epsilon }}_{i}\left(t\right)+G_{i}\left(\epsilon_{3_{i}},{\hat{w}}_{i}\right)\nu_{i}\left(\phi_{i}\right){\dot{\phi }}_{i}\left(t\right)$$

where,

$$A_{i}=\left[\begin{array}{cc} -\lambda_{1_{i}} & \omega_{i}\left(\phi_{i}\right){\dot{\phi }}_{i}\left(t\right)\\ -\omega_{i}\left(\phi_{i}\right){\dot{\phi }}_{i}\left(t\right) & -\lambda_{1_{i}} \end{array}\right]$$

$$G_{i}\left(\epsilon_{3_{i}},{\hat{w}}_{i}\right)=\left[\begin{array}{c} 2\sin^{2}\left(\frac{\epsilon_{3_{i}}}{2}\right)\\ \sin(\epsilon_{3_{i}}) \end{array}\right].$$

Notice that by Assumption 1, we obtain,

$$\begin{array}{c} \left|\right|G_{i}\left(\epsilon_{3_{i}},{\hat{w}}_{i}\right)\nu_{i}\left(\phi_{i}\right){\dot{\phi }}_{i}\left|\right|\leq \left|\right|G_{i}\left(\epsilon_{3_{i}},{\hat{w}}_{i}\right)\left||\right|\nu_{i}\left|\right|{\dot{\tau }}_{i}|\leq \beta_{i}\left|\epsilon_{3_{i}}\right|\bar{\nu_{i}}\mu_{i} \end{array}$$

for $\beta_{i}>0$. Therefore, $G_{i}\left(\epsilon_{3_{i}},{\hat{w}}_{i}\right)\nu_{i}\left(\phi_{i}\right){\dot{\phi }}_{i}$ is a fading exogenous signal for system (19) and tends to zero as $\epsilon_{3_{i}}\left(t\right)$ approaches the origin, despite the evolution of $\epsilon_{1_{i}}\left(t\right)$ and $\epsilon_{2_{i}}\left(t\right)$. Then, errors $\epsilon_{1_{i}}\left(t\right)$ and $\epsilon_{2_{i}}\left(t\right)$ converge to the origin according to the evolution of the disturbance-free system,

(20) $$\begin{array}{cc} {\dot{\bar{\epsilon }}}_{i}\left(t\right)= & A_{i}\left(t\right){\bar{\epsilon }}_{i}\left(t\right). \end{array}$$

For the time-varying system (20) consider a candidate Lyapunov function given as,

(21) $$V_{i}\left({\bar{\epsilon }}_{i}\right)=\frac{1}{2}\epsilon_{1_{i}}^{2}\left(t\right)+\frac{1}{2}\epsilon_{2_{i}}^{2}\left(t\right).$$

The time derivative of (21) produces,

(22) $${\dot{V}}_{i}\left({\bar{\epsilon }}_{i}\right)=-\lambda_{1_{i}}\left(\epsilon_{1_{i}}^{2}\left(t\right)+\epsilon_{2_{i}}^{2}\left(t\right)\right)=-2\lambda_{1_{i}}V_{i}\left(t\right)$$

that establishes global exponential stability of the errors $\epsilon_{1_{i}}\left(t\right)$ and $\epsilon_{2_{i}}\left(t\right)$. □

**Remark** **3.** *Note that the provided observer (12) exponentially converge depending on the values $\lambda_{ji}$, no matter what time delay is considered. Further, note that the convergence of the observer state to the delayed state of the i-th robot renders the delayed values of the desired velocities, that all together, provide the desired delayed path for the corresponding (i+1)-th robot.*

**Remark** **4.** *It should be pointed out that observer (12) prevents using an approximate estimation of the velocities of the i-th robot, for example, by means of the so-called dirty derivative, that could be an obstacle for the observer-based closed loop stability analysis, which is presented in the next section. Furthermore, the use of the observer does not require storing a large amount of data, while rendering robustness against noise and corrupted or missing data as the observer acts as a natural filter.*

**Remark** **5.** *Notice that Assumption 2 corresponds to a physical constraint for the leader robot in the formation, which allows having a feasible trajectory for the entire platoon that will satisfy the non-holonomic constraint, (2), as soon as convergence of the observation errors is achieved.*

## 4. Navigation Strategy

The platoon consists of a set of *n* robots that travel in chain formation as shown in Figure 2. To get the convergence of the state of the (i+1)-th robot to the state of the *i*-th observer, without loss of generality, it is assumed that the leader robot in the formation (i=1) is always in motion, this is stated at the following assumption.

**Assumption** **3.** *The input velocities of the leader robot (i=1) are so that,*

$$\lim_{t\to \infty }\nu_{1}\left(t\right)\neq 0\;\mathrm{or}\;\lim_{t\to \infty }\omega_{1}\left(t\right)\neq 0.$$

**Remark** **6.** *Related to Assumption 3, notice that the case $\nu_{1}\left(t\right)=\omega_{1}\left(t\right)=0$, force the overall formation to be in a standstill, a situation that is not relevant from a control formation point of view. On the other hand, the case $\nu_{1}\left(t\right)\neq 0$, $\omega_{1}\left(t\right)=0$ produces a motion in a straight line of the formation, and the case $\nu_{1}\left(t\right)=0$, $\omega_{1}\left(t\right)\neq 0$ produces a rotation of the leader robot at a point; thus, the desired chain formation does not make sense.*

For the navigation strategy, we take into account the kinematic model of the (i+1)-th robot

(23) $$\begin{array}{cc} {\dot{x}}_{i+1}\left(t\right) & =\nu_{i+1}\left(t\right)\cos\theta_{i+1}\left(t\right)\\ {\dot{y}}_{i+1}\left(t\right) & =\nu_{i+1}\left(t\right)\sin\theta_{i+1}\left(t\right)\\ {\dot{\theta }}_{i+1}\left(t\right) & =\omega_{i+1}\left(t\right). \end{array}$$

As mentioned earlier, the (i+1)-th robot will track the trajectory, delayed $\tau_{i}\left(t\right)$ units of time, of its precedent *i*-th robot that will be estimated by means of the *i*-th observer (12). Initially, we define the tracking errors between the state of the (i+1)-th robot and the *i*-th observer (12), as

(24) $$\begin{array}{c} e_{s_{1i}}\left(t\right)=x_{i+1}\left(t\right)-{\hat{w}}_{1_{i}}\left(t\right)\\ e_{s_{2i}}\left(t\right)=y_{i+1}\left(t\right)-{\hat{w}}_{2_{i}}\left(t\right)\\ e_{s_{3i}}\left(t\right)=\theta_{i+1}\left(t\right)-{\hat{w}}_{3_{i}}\left(t\right). \end{array}$$

To facilitate the analysis of the tracking errors, a change of coordinates in the form of (14) is considered, i.e.,

(25) $$\left[\begin{array}{c} e_{1_{i}}\left(t\right)\\ e_{2_{i}}\left(t\right)\\ e_{3_{i}}\left(t\right) \end{array}\right]=\left[\begin{array}{ccc} \cos\theta_{i+1}\left(t\right) & \sin\theta_{i+1}\left(t\right) & 0\\ -\sin\theta_{i+1}\left(t\right) & \cos\theta_{i+1}\left(t\right) & 0\\ 0 & 0 & 1 \end{array}\right]\left[\begin{array}{c} e_{s_{1i}}\left(t\right)\\ e_{s_{2i}}\left(t\right)\\ e_{s_{3i}}\left(t\right) \end{array}\right].$$

The time derivative of the errors (25) produces,

(26) $$\begin{array}{cc} {\dot{e}}_{1_{i}}\left(t\right)= & \nu_{i+1}\left(t\right)-{\dot{\hat{w}}}_{1_{i}}\left(t\right)\cos\theta_{i+1}\left(t\right)-{\dot{\hat{w}}}_{2_{i}}\left(t\right)\sin\theta_{i+1}\left(t\right)\\ \; & +\omega_{i+1}\left(t\right)e_{2_{i}}\left(t\right)\\ {\dot{e}}_{2_{i}}\left(t\right)= & {\dot{\hat{w}}}_{1_{i}}\left(t\right)\sin\theta_{i+1}\left(t\right)-{\dot{\hat{w}}}_{2_{i}}\left(t\right)\cos\theta_{i+1}\left(t\right)\\ \; & -\omega_{i+1}\left(t\right)e_{1_{i}}\left(t\right)\\ {\dot{e}}_{3_{i}}\left(t\right)= & \omega_{i+1}\left(t\right)-{\dot{\hat{w}}}_{3_{i}}\left(t\right). \end{array}$$

For the tracking error system (26) we now consider the virtual inputs,

(27) $$\begin{array}{cc} u_{1_{i}}\left(t\right)= & \nu_{i+1}\left(t\right)-{\dot{\hat{w}}}_{1_{i}}\left(t\right)\cos\left(\theta_{i+1}\left(t\right)\right)-{\dot{\hat{w}}}_{2_{i}}\left(t\right)\sin\left(\theta_{i+1}\left(t\right)\right)\\ u_{2_{i}}\left(t\right)= & \omega_{i+1}\left(t\right)-{\dot{\hat{w}}}_{3_{i}}\left(t\right) \end{array}$$

that in closed-loop produces the new representation,

(28) $$\begin{array}{cc} {\dot{e}}_{1_{i}}\left(t\right)= & u_{1_{i}}\left(t\right)+\omega_{i+1}\left(t\right)e_{2_{i}}\left(t\right)\\ {\dot{e}}_{2_{i}}\left(t\right)= & {\dot{\hat{w}}}_{1_{i}}\left(t\right)\sin\left(\theta_{i+1}\left(t\right)\right)-{\dot{\hat{w}}}_{2_{i}}\left(t\right)\cos\left(\theta_{i+1}\left(t\right)\right)\\ \; & -\omega_{i+1}\left(t\right)e_{1_{i}}\left(t\right)\\ {\dot{e}}_{3_{i}}\left(t\right)= & u_{2_{i}}\left(t\right). \end{array}$$

It is possible now, inspired by [25,27], to propose the nonlinear feedback,

(29) $$\begin{array}{cc} u_{1_{i}}\left(t\right)= & -k_{1i}e_{1_{i}}\left(t\right)\\ u_{2_{i}}\left(t\right)= & -k_{2i}\nu_{i}\left(\phi_{i}\right){\dot{\phi }}_{i}e_{2_{i}}\left(t\right)\frac{\sin(e_{3_{i}}\left(t\right))}{e_{3_{i}}\left(t\right)}-k_{3i}e_{3_{i}\left(t\right)}. \end{array}$$

Taking into account Equations (27) and (29), the actual feedback that solves the stabilization problem for the tracking errors (26) is written in the form,

(30) $$\begin{array}{cc} \nu_{i+1}\left(t\right)= & {\dot{\hat{w}}}_{1_{i}}\left(t\right)\cos\left(\theta_{i+1}\right)+{\dot{\hat{w}}}_{2_{i}}\left(t\right)\sin\left(\theta_{i+1}\right)-k_{1i}e_{1_{i}}\left(t\right)\\ \omega_{i+1}\left(t\right)= & {\dot{\hat{w}}}_{3_{i}}\left(t\right)-k_{2i}\nu_{i}\left(\phi_{i}\right){\dot{\phi }}_{i}e_{2_{i}}\left(t\right)\frac{\sin(e_{3_{i}}\left(t\right))}{e_{3_{i}}\left(t\right)}-k_{3i}e_{3_{i}}\left(t\right). \end{array}$$

The closed-loop system (26)–(30) is obtained as,

(31) $$\begin{array}{cc} {\dot{e}}_{1_{i}}\left(t\right)= & -k_{1i}e_{1_{i}}\left(t\right)+{\dot{\hat{w}}}_{3_{i}}\left(t\right)e_{2_{i}}\left(t\right)\\ \; & -k_{2i}\nu_{i}\left(\phi_{i}\right){\dot{\phi }}_{i}e_{2_{i}}^{2}\left(t\right)\frac{\sin(e_{3_{i}}\left(t\right))}{e_{3_{i}}\left(t\right)}-k_{3i}e_{2_{i}}\left(t\right)e_{3_{i}}\left(t\right)\\ {\dot{e}}_{2_{i}}\left(t\right)= & \nu_{i}\left(\phi_{i}\right){\dot{\phi }}_{i}\frac{\sin(e_{3_{i}}\left(t\right))}{e_{3_{i}}\left(t\right)}\left[e_{3_{i}}\left(t\right)+k_{2i}e_{2_{i}}\left(t\right)e_{1_{i}}\left(t\right)\right]\\ \; & -{\dot{\hat{w}}}_{3_{i}}\left(t\right)e_{1_{i}}\left(t\right)+k_{3i}e_{1_{i}}\left(t\right)e_{3_{i}}\left(t\right)+\psi_{i}\left(t\right)\\ {\dot{e}}_{3_{i}}\left(t\right)= & -k_{2i}\nu_{i}\left(\phi_{i}\right){\dot{\phi }}_{i}e_{2_{i}}\left(t\right)\frac{\sin(e_{3_{i}}\left(t\right))}{e_{3_{i}}\left(t\right)}-k_{3i}e_{3_{i}}\left(t\right) \end{array}$$

where,

(32) $$\psi_{i}\left(t\right)=\lambda_{1_{i}}\left[e_{w_{1i}}\left(t\right)\sin\left(\theta_{i+1}\right)-e_{w_{2i}}\left(t\right)\cos\left(\theta_{i+1}\right)\right]$$

and

$${\dot{\hat{w}}}_{3_{i}}\left(t\right)={\dot{\phi }}_{i}\left(t\right)\omega_{i}\left(\phi_{i}\right)+\lambda_{2_{i}}e_{w_{3i}}\left(t\right).$$

### Tracking Errors Convergence

The convergence properties of the navigation strategy are formally presented in the following lemma.

**Lemma** **2.** *Consider that Assumptions 2 and 3 are fulfilled and $k_{1i}$, $k_{2i}$, $k_{3i}>0$. Then, feedback (30) asymptotically stabilizes the origin of the tracking error system (26).*

**Proof.**  To see the convergence of the tracking errors in (31), notice first that the time-varying term $\psi_{i}\left(t\right)$ only depends on the observation errors $e_{w_{1i}}\left(t\right)$ and $e_{w_{2i}}\left(t\right)$ whose convergence was already proven, therefore $\psi_{i}\left(t\right)\to 0$ as $e_{w_{ji}}\left(t\right)\to 0$ independently of the evolution of the tracking errors $e_{j_{i}}$, and therefore, it can be considered as a fading exogenous signal. Because of this, the stability of the closed-loop system (31) can be established by analyzing the perturbation-free system (31) obtained by considering $\psi_{i}\left(t\right)=0$.

To show the stability of system (31) with $\psi_{i}\left(t\right)=0$, consider the candidate Lyapunov function,

(33) $$\begin{array}{c} V\left(e_{j_{i}}\right)=\frac{k_{2i}}{2}\left[e_{1_{i}}^{2}\left(t\right)+e_{2_{i}}^{2}\left(t\right)\right]+\frac{1}{2}e_{3_{i}}^{2}\left(t\right) \end{array}$$

with a time derivative given by,

(34) $$\begin{array}{cc} \dot{V}\left(e_{j_{i}}\right) & =k_{2i}\left[{\dot{e}}_{1_{i}}\left(t\right)e_{1_{i}}\left(t\right)+{\dot{e}}_{2_{i}}\left(t\right)e_{2_{i}}\left(t\right)\right]+{\dot{e}}_{3_{i}}\left(t\right)e_{3_{i}}\left(t\right)\\ \; & =-k_{1i}k_{2i}e_{1_{i}}^{2}\left(t\right)-k_{3i}e_{3_{i}}^{2}\left(t\right). \end{array}$$

Since $\dot{V}\left(e_{j_{i}}\right)\leq 0$, the system is stable. If Assumption 2 is satisfied, along the system solution, $e_{j_{i}}$ and thus ${\dot{e}}_{j_{i}}$ are bounded; this implies that the time derivative of $\dot{V}\left(e_{j_{i}}\right)$ is bounded and therefore it is uniformly continuous.

Invoking Barbalat’s Lemma [28], $\dot{V}\left(e_{j_{i}}\right)⟶0$. That is, $e_{1_{i}}\left(t\right)$ and $e_{3_{i}}\left(t\right)$ converge to zero.

From system (31) with $\psi_{i}\left(t\right)=0$, it is clear that,

(35) $$\begin{array}{cc} 0= & \omega_{i}\left(\phi_{i}\right){\dot{\phi }}_{i}\left(t\right)e_{2_{i}}\left(t\right)\\ 0= & -\nu_{i}\left(\phi_{i}\right){\dot{\phi }}_{i}k_{2i}\frac{\sin(e_{3_{i}})}{e_{3_{i}}\left(t\right)}e_{2_{i}}\left(t\right) \end{array}$$

because of the convergence of $e_{1_{i}}\left(t\right)$ and $e_{3_{i}}\left(t\right)$.

Notice that,

$$\lim_{e_{3_{i}}\left(t\right)\to 0}\frac{\sin e_{3_{i}}\left(t\right)}{e_{3_{i}}\left(t\right)}=1$$

and since ${\dot{\phi }}_{i}\left(t\right)=1-{\dot{\tau }}_{i}\left(t\right)$ can not be zero with a bounded $\tau_{i}\left(t\right)$; therefore, if Assumption 3 is satisfied, $e_{2_{i}}\left(t\right)$ will converge to the origin. □

**Remark** **7.** *It should be pointed out that in spite of the time-varying delay $\tau_{c_{i}}$, the tracking errors $e_{ji}$ always converge, and therefore the time delay $\tau_{i}$ converge to a constant value inside the region ${\bar{r}}_{c_{i}}\leq d_{i}\left(t\right)\leq r_{c_{i}}$. Outside this region, $\tau_{i}$ is constant as desired.*

## 5. Navigation Strategy Evaluation

The performance of the formation scheme presented in this paper was evaluated through numerical simulations and real-time experiments carried out by considering a Lemniscate-type path, as well as an oval track racing circuit for a group of four vehicles. For the sake of comparison, for the Lemniscate path, simulation and experimental results are presented, showing good correspondence with the obtained results. Meanwhile, for the oval track and due to a lack of space, only experimental results are shown. Nevertheless, since such track was intended to evaluate the performance of the controller when the angular velocity is zero and for the case of reference velocities discontinuities, the experimental results allow concluding the robustness of the proposed controller.

### 5.1. Lemniscate-Type Path

Since the first robot of the platoon can follow any trajectory produced by the action of bounded input velocities, to generate a specific trajectory to the formation, it will be considered a path obtained by input velocity signals defined in the form,

(36) $$\begin{array}{c} \begin{array}{cc} \nu_{1}\left(t\right)= & \sqrt{{\dot{x}}_{ref}^{2}\left(t\right)+{\dot{y}}_{ref}^{2}\left(t\right)}\\ \omega_{1}\left(t\right)= & \frac{{\ddot{y}}_{ref}\left(t\right){\dot{x}}_{ref}\left(t\right)-{\ddot{x}}_{ref}\left(t\right){\dot{y}}_{ref}\left(t\right)}{{\dot{x}}_{ref}^{2}\left(t\right)+{\dot{y}}_{ref}^{2}\left(t\right)} \end{array} \end{array}$$

where $x_{ref}\left(t\right)$ and $y_{ref}\left(t\right)$ correspond to the X−Y coordinates evolution of the desired reference path.

To generate a Lemniscate-type trajectory, the leader robot is fed with the linear ($\nu_{1}$) and angular ($\omega_{1}$) velocities given by (36) where it is considered,

(37) $$\begin{array}{ccc} x_{ref}\left(t\right) & = & -a\cos(pt)\\ y_{ref}\left(t\right) & = & b\sin(2pt) \end{array}$$

with a=0.8, b=0.6 and $p=\frac{2\pi }{50}$. This path involves orientation and velocity changes that influence the relative distance between the robots, allowing us to evaluate the effectiveness of the time-varying spacing policy presented in this paper.

For all the experiments and simulations, unless otherwise specified, the spacing policy (3) and (4) considers the parameters specified in Table 1 for i=1,2,3.

### 5.2. Numerical Evaluation in a Simulation Frame (NE)

To carry out the numerical evaluation, the gains used by the observers (12) were $\lambda_{1_{i}}=\lambda_{2_{i}}=0.5$ and $\lambda_{3_{i}}=0.3$ for i=1,2,3, and the gains for the control laws (30) of the follower robots were set to $k_{1_{i}}=0.4$, $k_{2_{i}}=0.1$, $k_{3_{i}}=0.25$ for i=2,3,4. The initial conditions of the mobile robots (i=1,2,3,4) and the delayed observers (i=1,2,3) are shown in Table 2.

For the spacing policy (3) and (4), to show the difference between the time-varying strategy developed in this work and the previous fixed-time gap strategy [23,24], for the second robot i=2 in the formation, it is assumed that for all *t*, $\tau_{c_{i}}=0$ and $t_{s_{1}}=2\left[s\right]$, thus, it performs as with a constant time gap.

To numerically show the robustness properties of the observer, two different disturbances were introduced to Robot 2 in the formation. The first one, at t=40 s considers a change on the position of the coordinate $x_{2}\left(t\right)$ from its actual value to $x_{2}\left(t\right)=-0.2$ m, emulating an instantaneous displacement on the robot position. The second disturbance considers a failure on the measurement of $x_{2}\left(t\right)$ adding 0.1 m to its actual value for the period of time 80≤t≤81.

**Figure 4:**
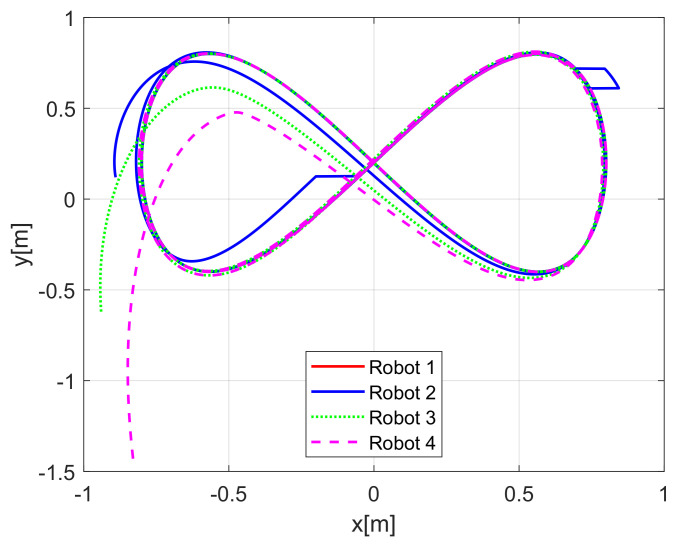
(NE) Mobile robots time evolution.

**Figure 5:**
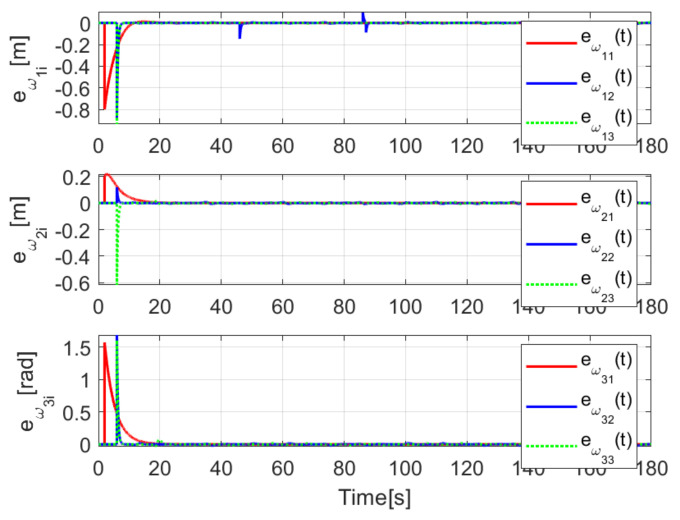
(NE) Observation errors $e_{w_{1_{i}}}\left(t\right)$, $e_{w_{2_{i}}}\left(t\right)$, $e_{w_{3_{i}}}\left(t\right)$.

Figure 4 shows the time evolution of the set of robots in the *X*-*Y* plane, where the effects of the introduced disturbances are clearly shown. It is evident how the time convergence to the delayed leader trajectory depends on the order of the mobile robot on the chain formation, it is also observed how the effects of the considered disturbance in Robot 2 are diminished for the remaining robots along the chain. As expected by the involved delays, the observations errors $e_{w_{1_{i}}}\left(t\right)$, $e_{w_{2_{i}}}\left(t\right)$, $e_{w_{3_{i}}}\left(t\right)$ displayed in Figure 5 converge, which also depend on the order of the robots in the formation and the considered disturbances are adequately filtered.

**Figure 6:**
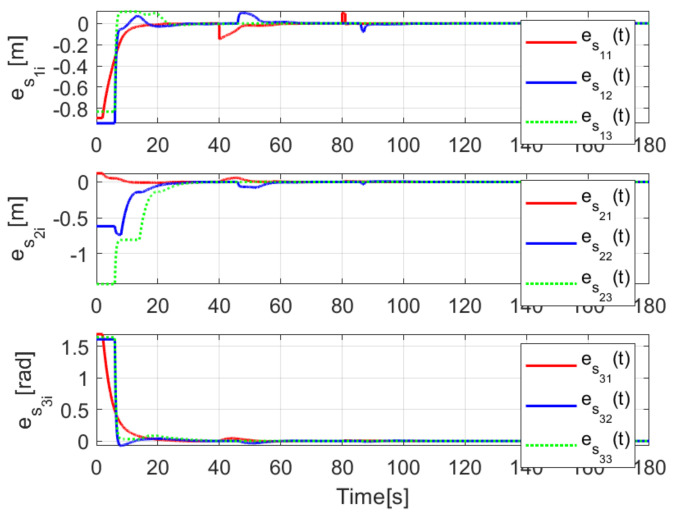
(NE) Tracking errors $e_{s1_{i}}\left(t\right)$, $e_{s2_{i}}\left(t\right)$, $e_{s3_{i}}\left(t\right)$.

The convergence of the tracking errors $e_{s1_{i}}\left(t\right)$, $e_{s2_{i}}\left(t\right)$, $e_{s3_{i}}\left(t\right)$ is shown in Figure 6, where it is evident that they converge, once the observation errors have reached the origin, as expected by the preceding developments. The introduced disturbance affecting Robot 2 are appropriately diminished for Robots 3 and 4.

**Figure 7:**
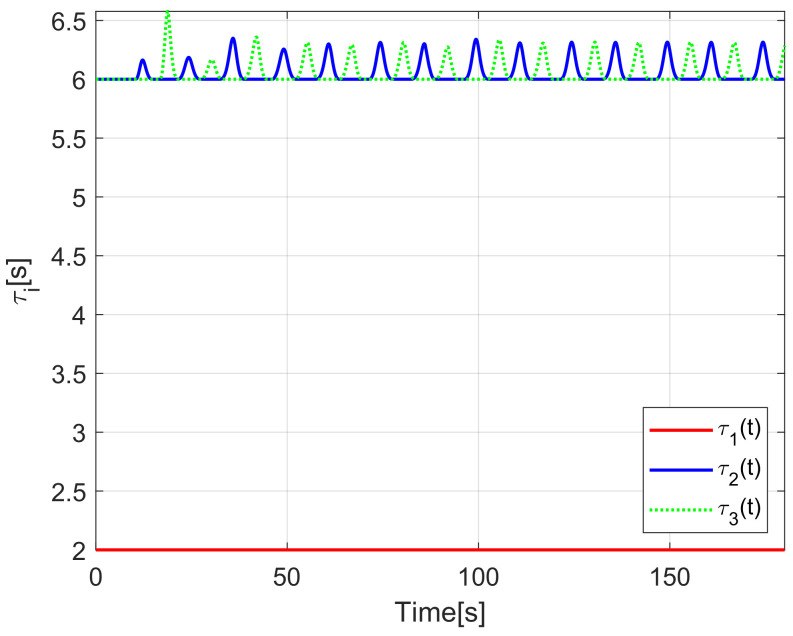
(NE) Time-varying gap $\tau_{i}\left(t\right)$.

**Figure 8:**
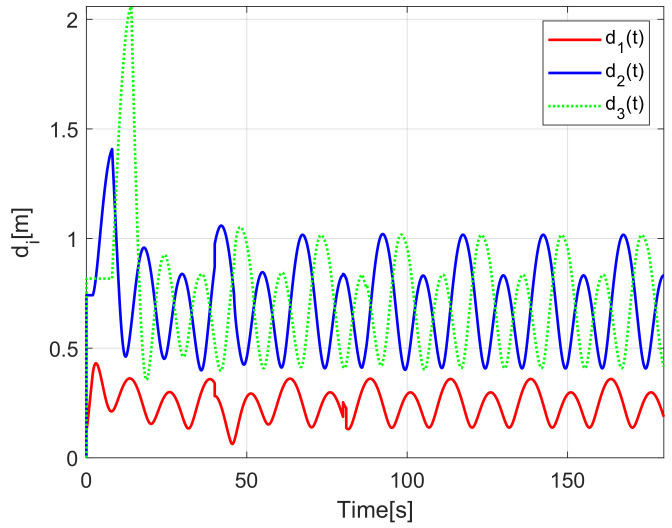
(NE) Relative distances $d_{i}\left(t\right)$.

**Figure 9:**
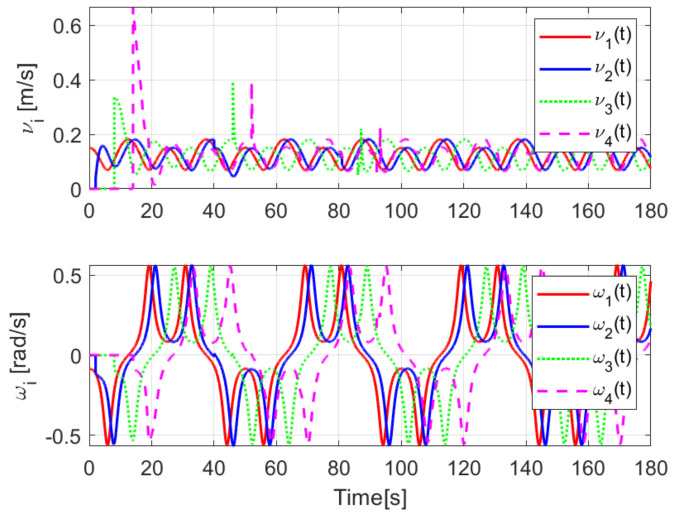
(NE) Control signals $\nu_{i}\left(t\right)$, $\omega_{i}\left(t\right)$.

The evolution of the time-varying gap $\tau_{i}\left(t\right)$ is shown in Figure 7, while the relative distances $d_{i}\left(t\right)$ obtained from the actual position of each pair of successive robots on the formation are presented in Figure 8. Finally, the control signals $\nu_{i}\left(t\right)$, $\omega_{i}\left(t\right)$ for each robot are depicted in Figure 9.

**Figure 10:**
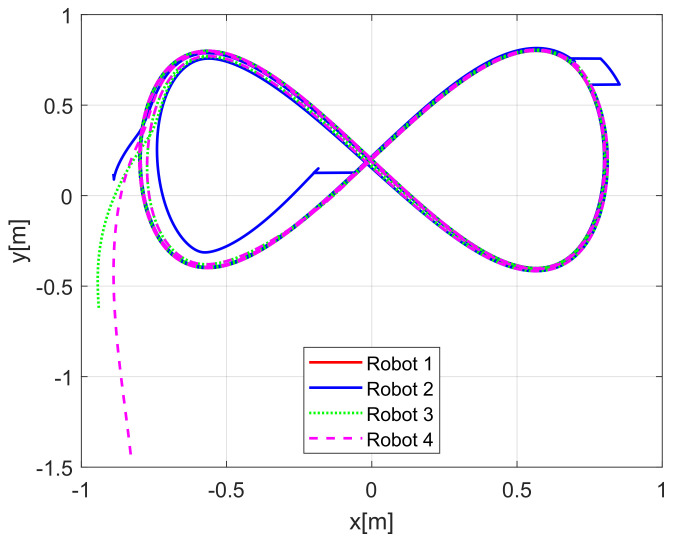
(NE) Improved tracking evolution under disturbances.

**Figure 11:**
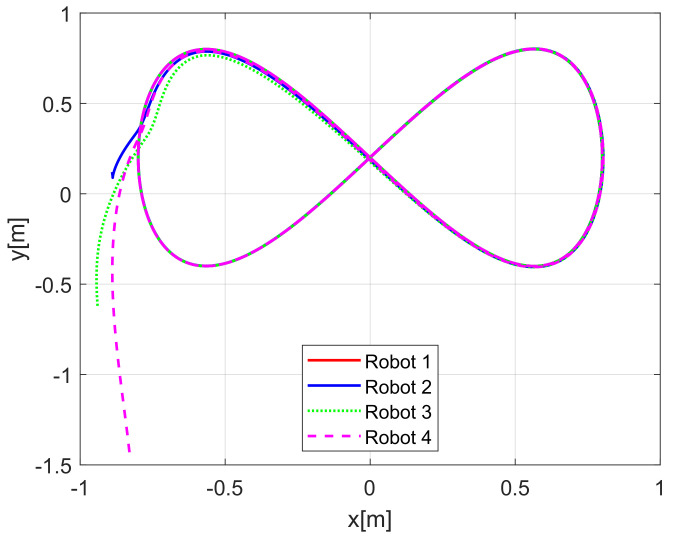
(NE) Improved disturbance-free tracking evolution.

In the above numerical evaluation, the considered gains were chosen according to the velocity characteristics of the physical mobile robots used on the real-time experiments presented in the next subsection. To show a better convergence of the tracking errors, and the effects of tuning gains, the gains were set as $\lambda_{1i}=\lambda_{2i}=2$ for i=1,2,3 for the observers and $k_{11}=8$, $k_{21}=10$, $k_{31}=3$ and $k_{1i}=8$, $k_{2i}=10$, $k_{3i}=3$ for i=2,3 for the control law. For the new set of gains, the trajectories of the robots on the X−Y plane are shown in Figure 10 for the perturbed case, and in Figure 11 for a disturbance-free experiment.

### 5.3. Real-Time Experiment (RTE)

In order to test the proposed control law, an experimental platform was used to perform two real-time experiments. This platform consists of three differentially driven mobile robots *TurtleBot3* type *Burger*, equipped with a *Raspberry Pi Model B* and wireless communication, with one virtual robot used as a leader in the formation. The physical robots were used as followers. As mentioned in the control strategy, the virtual robot, under bounded input velocities, provides the trajectory that the follower robots should track. The physical robots have four passive markers (reflective) that are used to obtain their geometrical centroid and in this way compute its position and orientation by means of the localization system.

The vision-based indoor localization system consists of 12 *Flex-13* cameras with an image resolution of 1280×1024, and 120 frames per second (FPS), it was assembled on the roof and produces a vision-working area of 20 m${}^{2}$; these cameras have a LED IR ring and a image sensor, the IR light is emitted and the passive markers reflect this light to the camera image sensor, in this form, the position and orientation was obtained for all the robots by using the software *Motive*; similar to emulate a GPS localization system. This information was sent to a personal computer where the data are used to obtain the delayed time-varying observers and the control law signals for all the robots; when all the control laws were obtained, the signals were sent by wireless communication through a VRPN (virtual reality peripheral network) and software ROS (robot operating system) that serves as a link between robots and devices.

**Figure 12:**
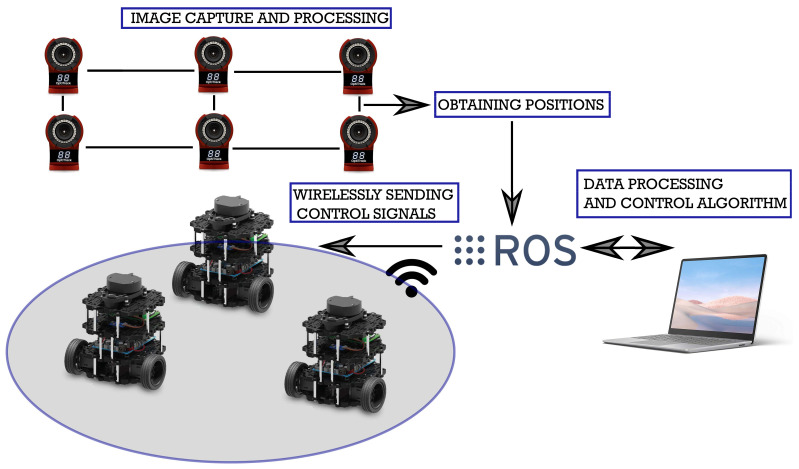
Experimental platform.

To consider a set of four robots in the chain formation, as in the case of the numerical study (NE) presented in Section 5.1, without lost of generality, it is assumed that the leader robot (i=1) is a virtual robot that will generate the trajectory that the remaining robots have to follow with specific time delay. In this way, the virtual leader robot is followed by three *Turtlebot3* robots forming a string of four vehicles that requires the use of three delayed observers. The distribution of the experimental platform is illustrated in Figure 12.

The gains used for the observers (12), and the gains for the control laws (30) are equal to the ones used for the numerical evaluation (NE) presented in Section 5.1.

The first real-time experiment corresponds to the Lemniscate-type path, for whose results are compared to the simulated ones presented in Section 5.1; this case would be referred as RTE-a. The second case involves an oval track racing, that includes discontinuous velocity changes of the mobile robots, when entering and leaving a curve path; this case would be referred as RTE-b.

#### 5.3.1. Lemniscate-Type Path Real-Time Experiment (RTE-a)

For comparison purposes, the first real-time experiment corresponds to the Lemniscate path given by (36), with the same parameters considered in the numerical simulation scenery presented in Section 5.1. The initial conditions of the mobile robots (i=1,2,3,4) and the delayed observers (i=1,2,3) are shown in Table 3. The spacing policy is determined by Table 1.

**Figure 13:**
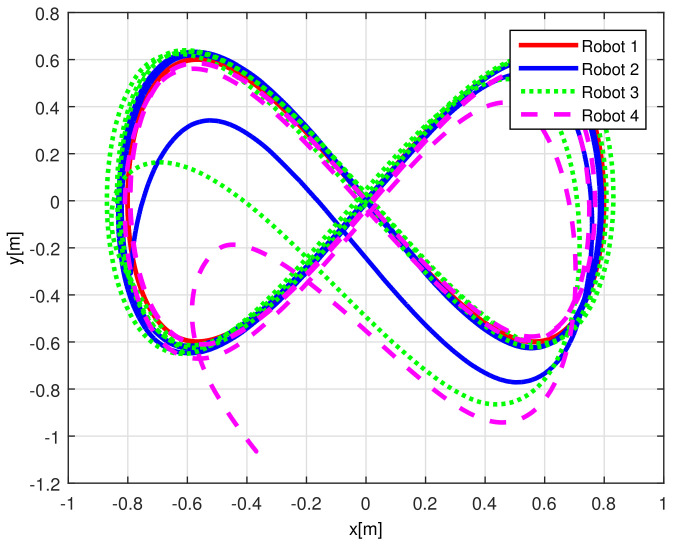
(RTE-a) Mobile robots time evolution, Lemniscate path.

Figure 13 displays the evolution carried out by the mobile robots at the *X*-*Y* plane, where the convergence of the vehicles to the desired path is obtained under the consideration of non-null initial conditions errors.

**Figure 14:**
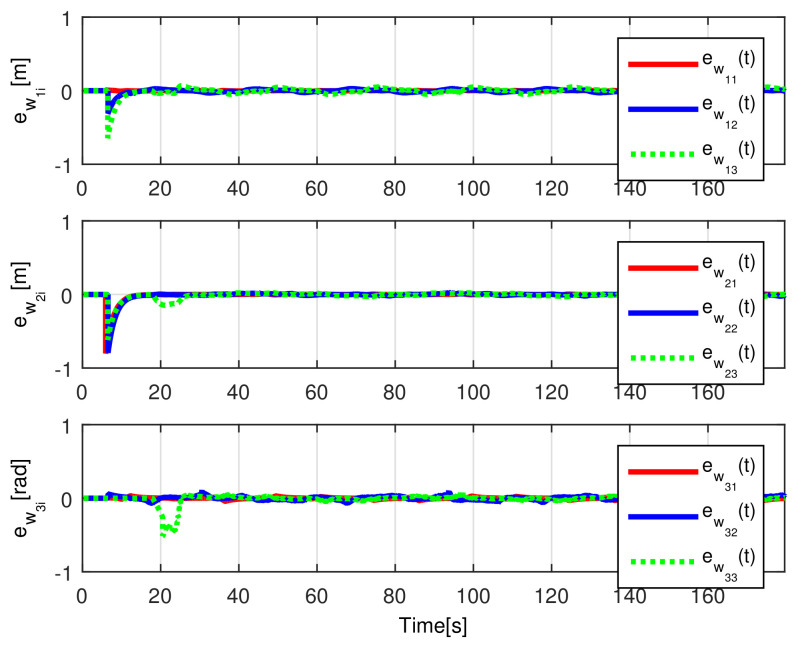
(RTE-a) Observation errors $e_{w_{1_{i}}}\left(t\right)$, $e_{w_{2_{i}}}\left(t\right)$, $e_{w_{3_{i}}}\left(t\right)$, Lemniscate path.

**Figure 15:**
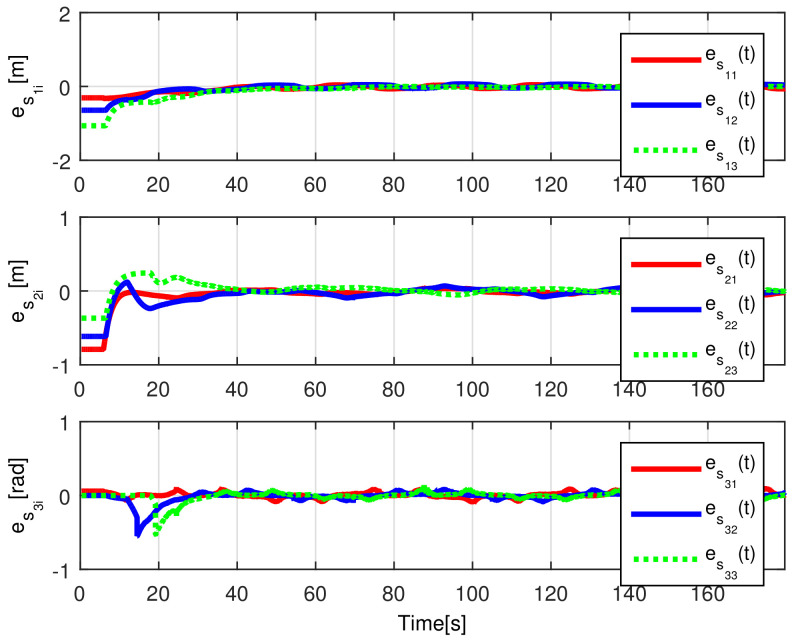
(RTE-a) Tracking errors $e_{s1_{i}}\left(t\right)$, $e_{s2_{i}}\left(t\right)$, $e_{s3_{i}}\left(t\right)$, Lemniscate path.

The convergence of the observation errors $e_{w_{1_{i}}}\left(t\right)$, $e_{w_{2_{i}}}\left(t\right)$, $e_{w_{3_{i}}}\left(t\right)$ is depicted in Figure 14, where after a transient period the observers states converge to the delayed trajectory of their respective *i*-th robot. The tracking position errors evolution is shown in Figure 15, it is evident how $e_{s1_{i}}\left(t\right)$, $e_{s2_{i}}\left(t\right)$, $e_{s3_{i}}\left(t\right)$ converge to the origin.

**Figure 16:**
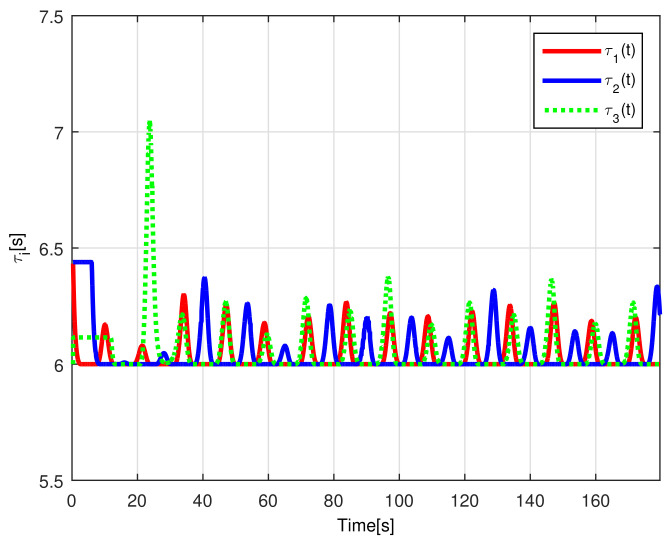
(RTE-a) Time-varying gap $\tau_{i}\left(t\right)$, Lemniscate path.

**Figure 17:**
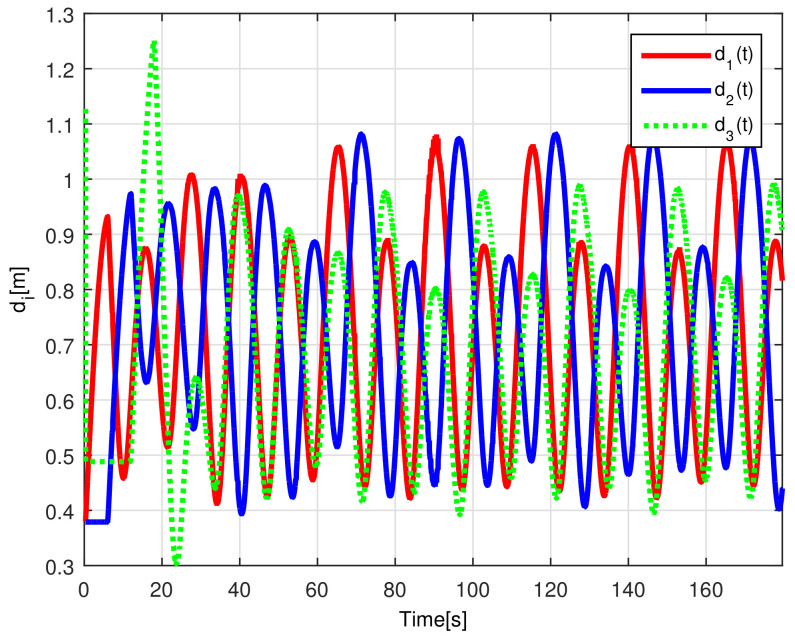
(RTE-a) Relative distances $d_{i}\left(t\right)$, Lemniscate path.

The time-varying gap $\tau_{i}\left(t\right)$ for i=1,2,3 is shown in Figure 16, notice that the magnitude of $\tau_{i}\left(t\right)$ increases only when the distance between the *i*-th and the (i+1)-th robot is less than the desired safe distance $r_{c_{i}}$, thus, increasing the physical distance $d_{i}\left(t\right)$ between the robots to avoid getting too close to each other, see Figure 17, where the time evolution of $d_{i}\left(t\right)$ for i=1,2,3 is depicted. In this case, the evolution of the distance $d_{i}\left(t\right)$ is measured by the *Optitrack* vision positioning system. Note that the time-varying gap activates at transients and at the curve sections of the desired path, where the trailing distance decreases, but such distance is smaller than the Euclidean distance that triggers the time-varying gap, thus rendering a conservative collision avoidance action.

**Figure 18:**
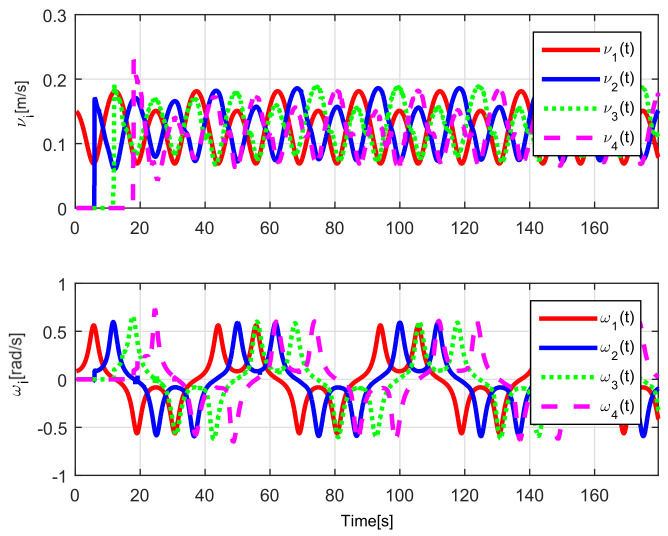
(RTE-a) Control signals $\nu_{i}\left(t\right)$, $\omega_{i}\left(t\right)$, Lemniscate path.

The evolution of the input signals $\nu_{i}\left(t\right)$, $\omega_{i}\left(t\right)$ for i=1,2,3,4 is shown in Figure 18, allowing to conclude bounded and continuous control actions.

#### 5.3.2. Oval Track Racing Real-Time Experiment (RTE-b)

For the second experimental test, we considered that the virtual leader robot follows a reference trajectory that describes a racing oval track. This trajectory was built by means of the combination of straight paths,

$$x\left(t\right)=gt,\;y\left(t\right)=br_{c}$$

and curve segments,

$$x\left(t\right)=bh+r_{c}\cos\left(a+pt\right),\;y\left(t\right)=k+r_{c}\sin\left(a+pt\right)$$

where $r_{c}=0.5,h=0.8,k=0$ and $p=\frac{\pi }{12}$. Some parameters change according to the segment of the path, $a=-\frac{\pi }{2},\frac{\pi }{2}$, b=1,−1, and g=0.08,0.13,0.16.

Note that the fact that the trajectory is sectionally designed implies that there will be discontinuous points in the velocities of the leader robot (i=1) that will be acting as a velocity disturbance for the chain of robots showing in this way the benefits of the observers. The initial conditions are shown in Table 4 and the gains used for the observers are $\lambda_{1_{i}}=\lambda_{2_{i}}=0.5$ and $\lambda_{3_{i}}=0.3$ for i=1,2,3. For the control laws, the gains are $k_{1_{i}}=0.1,k_{2_{i}}=0.15,k_{3_{i}}=0.5$ for i=1,2,3. The parameters for the spacing policy are also given by Table 1 were $t_{s_{i}}$ is adjusted to $t_{s_{i}}=5\left[s\right]$.

**Figure 19:**
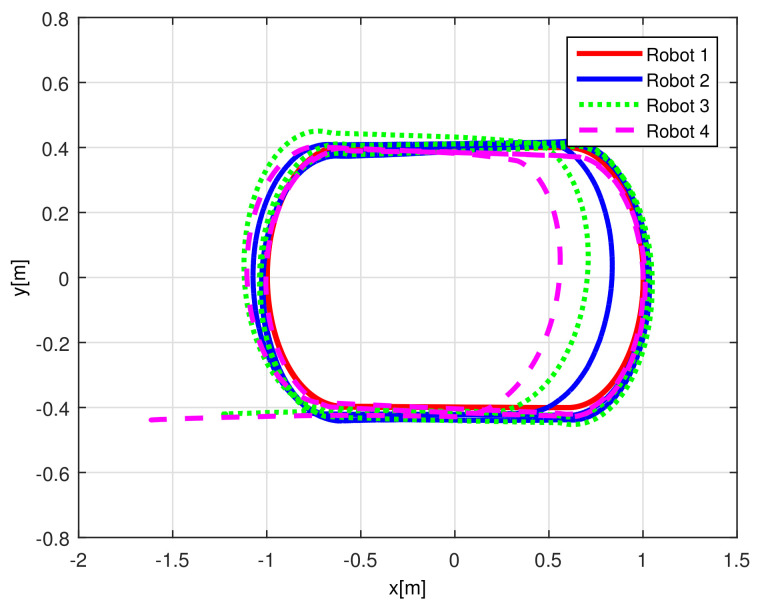
(RTE-b) Mobile robots time evolution, oval track racing.

Figure 19 displays the trajectories performed by each robot at the X−Y plane.

**Figure 20:**
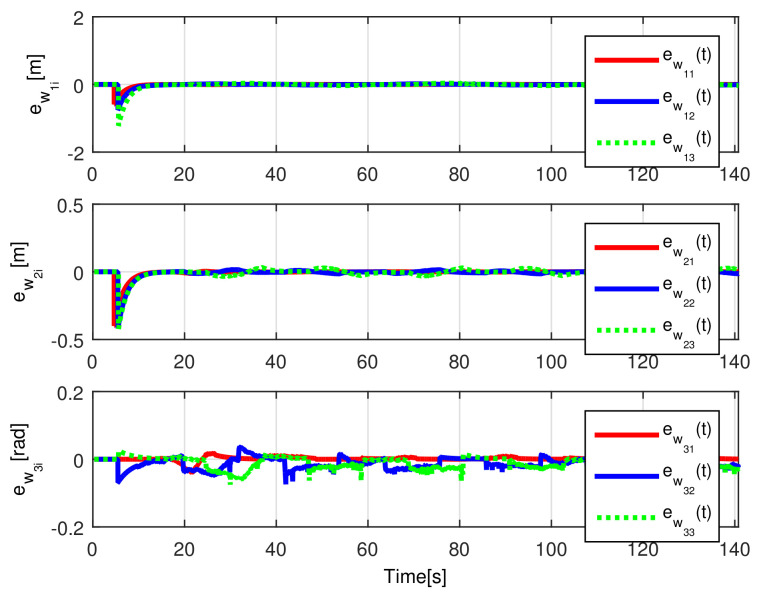
(RTE-b) Observation errors $e_{w_{1_{i}}}\left(t\right)$, $e_{w_{2_{i}}}\left(t\right)$, $e_{w_{3_{i}}}\left(t\right)$, oval track racing.

**Figure 21:**
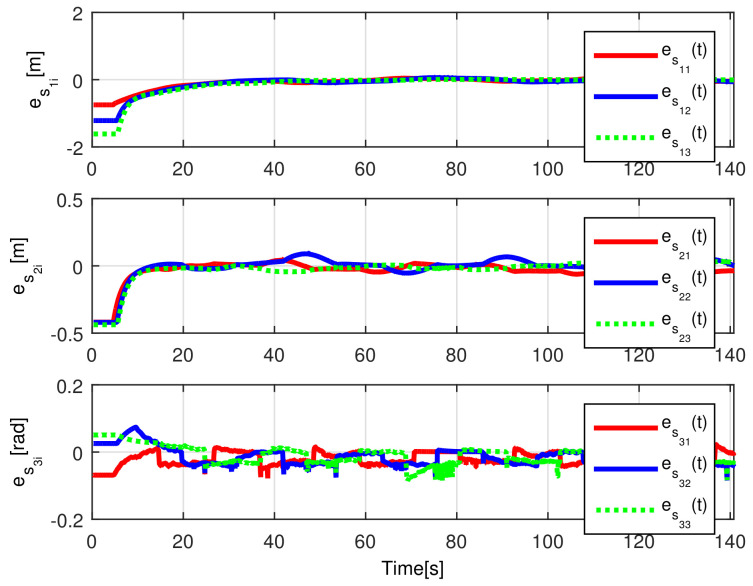
(RTE-b) Tracking errors $e_{s1_{i}}\left(t\right)$, $e_{s2_{i}}\left(t\right)$, $e_{s3_{i}}\left(t\right)$, oval track racing.

The observation errors $e_{w_{1i}}\left(t\right),e_{w_{2i}}\left(t\right),e_{w_{3i}}\left(t\right)$ are depicted in Figure 20, where their convergence is clearly shown, while the tracking position errors $e_{1_{i}}\left(t\right),e_{2_{i}}\left(t\right),e_{3_{i}}\left(t\right)$ are shown in Figure 21.

**Figure 22:**
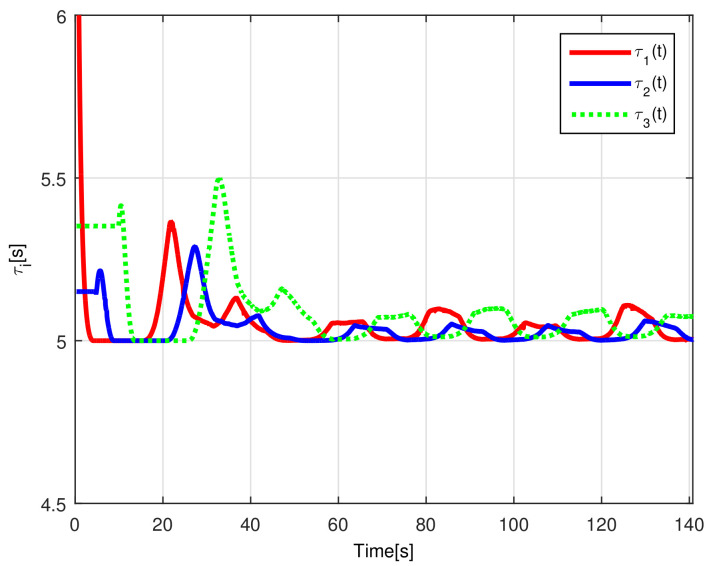
(RTE-b) Time-varying gap $\tau_{i}\left(t\right)$, oval track racing.

**Figure 23:**
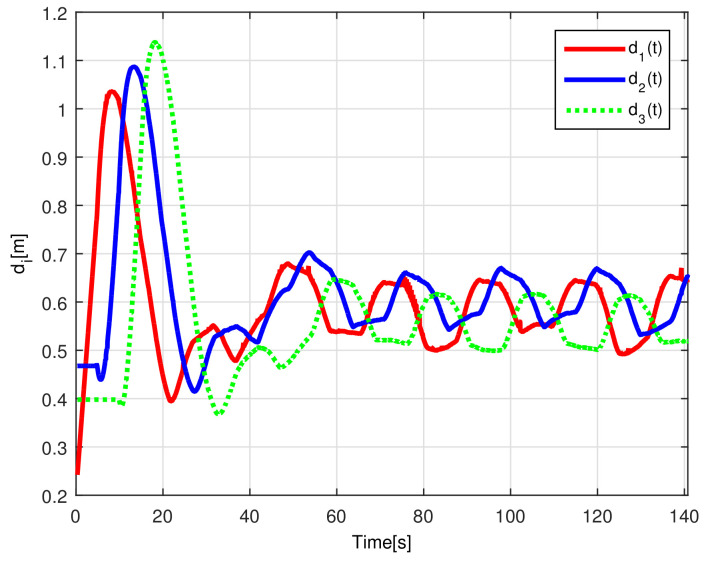
(RTE-b) Relative distances $d_{i}\left(t\right)$, oval track racing.

Figure 22 shows the time-varying gaps between robots, while Figure 23 presents the relative distances between the robots. Note that when the robots enter the curve, the relative distance between successive robots decreases and the time-varying gap grows, yielding a safe trailing distance.

**Figure 24:**
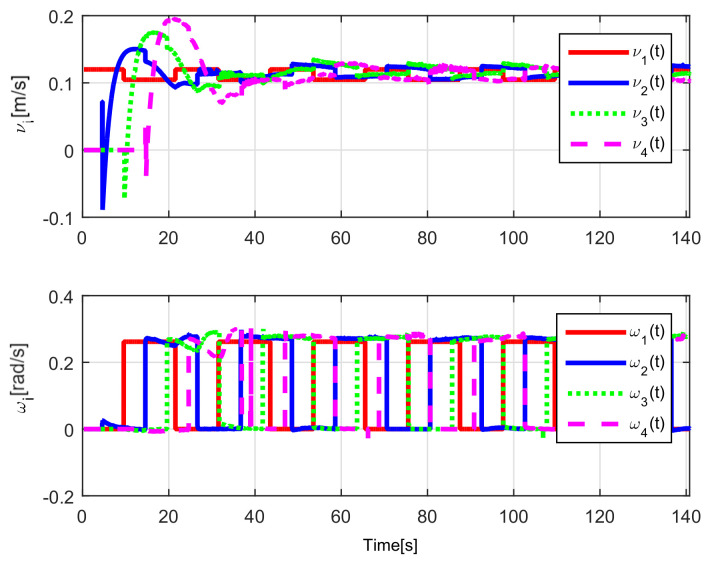
(RTE-b) Control signals $\nu_{i}\left(t\right)$, $\omega_{i}\left(t\right)$, oval track racing.

Finally, the control signals are shown in Figure 24. Note that the considered trajectory implies that the angular velocity will be zero when the convoy travels on the straight line, and that the linear velocity decreases during the curve, causing the robots to come closer to each other.

## 6. Results Discussion

At the introduction of the article, the state-of-the-art approaches were established for platoon formation strategies, as well as, for leader–follower setups. While designing the proposed time-varying spacing policy, a comparison to constant spacing policy was carried out, concluding that keeping constant distance and bearing angle between each pair of successive vehicles hinders the follower robots’ ability to perform the same path as described by the leader robot, mainly at curves. From these comparisons, it was evident that a varying distance and bearing angle are required at a curve. A similar comparison was performed with respect to time-based spacing policies, concluding that the space headway created by the separation time may render collisions when the translational velocity is small or tends to zero. However, for the sake of space, these comparison studies are not presented.

When comparing the simulated (NE) results, Section 5.1, and real-time experiments for the Lemniscate path (RTE-a), Section 5.3.1, we note that convergence of the observation errors is rather faster than the tracking errors, as established from the stability analysis, since the observations errors present exponential convergence, Lemma 1, while the tracking errors converge asymptotically, Lemma 2. In contrast, it can be seen that the time-varying gap and the associated relative distance ensures no collision between the mobile robots, preserving a minimum safe distance. Finally, as to show robustness, when sudden perturbations at positions and orientation are introduced, simulating lost or failure of sensors measurements, or communication channel problems, it can be seen that the observers filtered the peak changes on measurements values, and smoothness of the signals is propagated through the mobile robots chain.

Note that by Assumptions 2, 3, and Remark 6, some properties are required for the translation and rotation leader velocities, these constraints are used to prove stability and convergence properties of the observation and tracking errors. However, in real practice scenarios, rotational velocity can be zero, as when moving in a straight line; however, connecting a straight path with a curved one implies discontinuities at the rotational velocity. Thus, in order to show that, even in these scenarios, the proposed controller behaves appropriately and convergence properties are kept, the oval path experiment of Section 5.3.2 was tested. Note that although the mobile robots presents discontinuities, see Figure 24, the stability and convergence properties of the observation and tracking errors is preserved, thus, it can be concluded that the observers help filtering such discontinuities and render smooth tracking trajectories for the platoon formation, and this filter behavior is propagated through the mobile robot chain.

**Remark** **8.** *It is important to point out some drawbacks of the proposed time-varying gap formation strategy. First of all, it should be pointed out that the present chain strategy does not consider a specific obstacle avoidance strategy since at the transient response, when the tracking errors have not converged, there is a possibility of collision among the vehicles at the platoon; this risk is eliminated when the agents have converged to the desired trajectories due to the time-varying strategy. We also note that, even when the individual control of each robot allows the possibility to move backwards, the chain formation does not allow this situation since the leader trajectory has to be followed by all members of the formation. Backward movements could be possible in the case of a time-varying topology, allowing the last robot to become the new leader of the formation.*

## 7. Conclusions

In this work, a control scheme for a platoon of mobile robots with a time-varying spacing policy, based on an input-varying-delay observer that estimates the delayed trajectory of the (*i*)-th robot, which should be considered as a desired path for the (i+1)-th robot, was developed. The time-varying gap between each pair of successive robots is computed by means of a smooth function $\tau_{c_{i}}$ activated on an influence zone that depends on the distance between robots (*i*)-th and (i+1)-th.

It is formally shown, based on a Lyapunov stability analysis, how the estimation and the tracking errors associated with the chain formation of the vehicles converge to the origin, preserving the formation of the vehicles along any trajectory described by the leader robot in the workspace, due to bounded input velocity signals. The proposed formation strategy shows that when the robots approach each other in a slowdown velocity scenario, the time-varying gap raises its magnitude to increase the distance between each pair of successive robots at the platoon, avoiding collisions among them, rendering an efficient collision-free formation strategy.

Real-time experiments are in line with the simulation results, supporting the convergence properties that were obtained by the stability analysis over the estimation and tracking errors. The robustness benefits of the considered observer are shown by numerical and experimental results on a Lemniscate-path type and an oval track race example, showing good performance of the proposed formation strategy, allowing us to conclude some robustness properties of the platooning strategy.

It should be highlighted that the present strategy considers the complete kinematic model of the vehicles and not only a reduction model in one dimension or a punctual mass robot, as is usual in the literature. It is also important to mention that all the robots in the formation converge to the same trajectory generated by the platoon leader robot, and the fact that the considered delay observer acts as a natural filter for possible external or measurement disturbances. Finally, it is important to mention that the real-time experiments were carried out in a controlled environment; in a future work, we should consider the same formation problem for an outdoor experiment by adding onboard cameras to the follower robots, which could provide the relative distance and angles between the robots. Further, an obstacle avoidance strategy should be considered in a general solution of this formation problem.

## Figures and Tables

**Table 1:** Spacing policy parameters.

	$\alpha_{i}$	$r_{c_{i}}\left[m\right]$	${\bar{r}}_{c_{i}}\left[m\right]$	$t_{s_{i}}\left[m\right]$
Robot (i+1)	0.6	0.7	0.1	6

**Table 2:** Test 1: Initial conditions for the numerical evaluation (NE).

	*x* [m]	*y* [m]	θ[rad]
Robot 1	−0.8	0.2	π/2
Robot 2	−0.89	0.12	0.12+π/2
Robot 3	−0.94	−0.62	0.04+π/2
Robot 4	−0.83	−1.43	0.07+π/2
Observer i	0	0	0

**Table 3:** Initial conditions for Lemniscate-type path real-time experiment (RTE-a).

	*x* [m]	*y* [m]	θ[rad]
Robot 1	−0.8	0	π/2
Robot 2	−0.8	−0.3	π/2
Robot 3	−0.64	−0.6	−0.25+π/2
Robot 4	−0.36	−1.06	−0.41+π/2
Observer i	0	0	0

**Table 4:** Initial conditions for oval track racing real-time experiment (RTE-b).

	*x* [m]	*y* [m]	θ[rad]
Robot 1	−0.4	−0.6	0
Robot 2	−0.41	−0.75	0.06
Robot 3	−0.43	−1.2	0
Robot 4	−0.44	−1.6	0.03
Observer i	0	0	0

## Data Availability

Not applicable.
